# Inflammation in low back pain may be detected from the peripheral blood: suggestions for biomarker

**DOI:** 10.1042/BSR20160187

**Published:** 2016-08-05

**Authors:** Yong Li, Jun Liu, Zong-zhi Liu, Da-peng Duan

**Affiliations:** * Department of Orthopedics, Shaanxi Provincial People's Hospital, Xi'an, Shaanxi 710068, China

**Keywords:** biomarker, inflammation, low back pain, molecular mechanism, transcription

## Abstract

Biomarker for prediction of development of low back pain, and disease progression in chronic conditions are virtually non-existent. In the present study, we examined evidence of inflammation in the peripheral blood and demonstrated significant changes in neuroinflammation markers in subjects with chronic low back pain in comparison with control subjects. The present study was performed using peripheral blood from subjects with chronic low back pain and age-matched control subjects. Western blotting, real-time RT-PCR, cell culture and *in vitro* assays were incorporated to perform the current study. We obtained evidence that the balance between proinflammatory and anti-inflammatory cytokines is misaligned, with decrease in interleukin-10 (IL-10) expression and increase in interleukin-6 (IL-6) expression. Furthermore, we demonstrated increase in CD16 monocyte expression. Cells were cultured under differential conditions to generate M1/M2 macrophages. In the macrophages, opioid secretory capacity was shown to be diminished. Finally, Dragon (repulsive guidance molecule b, RGMb) expression was shown diminished in M1 macrophages, which serves as a key transcriptional inhibitor of IL-6 expression. These biochemical and cellular alterations in chronic low back pain can serve as potential biomarkers for assessing disease initiation, intensity and progression.

## Abstract (precis)

The results of our study provides novel insights into the mechanisms of low back pain, as well as provide suggestions of the possibility that these assays may be used as biomarkers for low back pain.

## INTRODUCTION

Low back pain is a distressing complaint in primary care clinics and also neurology/orthopaedics and varied other consults [1,2]. The basis for low back pain is not well understood [3–6]. Though imaging studies have been performed and characterized the numerous pleomorphic presentations of lower vertebral changes in low back pain [7], there are no peripheral biomarkers that can predict predisposition of disease or disease progression.

In the present study, we hypothesized whether peripheral blood may be used to assay useful pertinent pathophysiologic information pertaining to the mechanism of low back pain. We based our hypothesis on the fact that transformed cells as well as few peripheral blood cells have the capability of secreting endogenous opioid-like peptides and suppress general pain conditions [8,9], and that these regulatory mechanisms may be disrupted in chronic low back pain.

We performed the study in two phases: we obtained peripheral blood and assayed pro- and anti-inflammatory cytokines that play major roles in creating lineages to induce or suppress inflammation, including neuro-inflammation. Thereafter, we tried to culture the peripheral blood cells and differentiate into specific sets of macrophages by controlled growth factor stimulation over pre-established duration of time. We examined the expression of opioid-like peptides released in these macrophages. This was an in-situ method to establish a model of examining the neuro-immune interfacing environment that possibly occurs in the dorsal root ganglion (DRG). For example, there are anecdotal reports of blood cells recruited to the DRG regions and thereafter modulating nociceptive processing modalities by secretion of endogenous opioids [10,11]. The results of our study provide novel insights into the mechanisms of low back pain, as well as provide suggestions of the possibility that these assays may be used as biomarkers for low back pain.

## MATERIALS AND METHODS

### Patient blood samples

All experiments were performed after obtaining explicit institutional permission from the Human Experiments Committee and performed according to established ethical guidelines for experimentation with human samples via Helsinki declaration. Blood samples were obtained and processed for cellular and soluble biomarkers for chronic low back pain. Thirty-five subjects with low back pain (aged 45–70), 35 subjects with upper back pain (aged 45–70), and similar age-matched control subjects were incorporated in the present study. The ranges of the body weights were comparable. Blood was obtained in the AM time for all subjects. All blood was treated immediately for isolation of blood cells and separation of plasma.

### Separation of peripheral blood mononuclear cells

Veniclysis was performed to obtain blood samples. These were collected into EDTA-coated vacutainers. After centrifugation, the buffy coat was carefully aspirated and resuspended in Roswell Park Memorial Institute (RPMI)-1640 media. This aspirate was overlaid on to Histopaque-1077 (Sigma) for further steps of isolation of peripheral blood mononuclear cells (PBMCs) by gradient centrifugation. The plasma was aliquotted in equal proportions. This was refrigerated and stored until further assays for cytokine assay.

### Flow cytometry to assess monocyte-specific populations

The separated PBMCs were washed and thereafter added to PBS containing varied combinations of fluorescent-conjugated antibodies. The specific antibodies were targeted for CD4 [fluorescence-activated cell sorter (FITC)] and CD8 [phycoerythrin-cyanine5 (PE-Cy5)] for T-lymphocytes, CD19 (PE) for B-lymphocytes and CD14 [allophycocyanin (APC)] and CD16 (FITC) for the peripheral monocytes. The blood cells were persistently incubated in staining mixtures on ice and in complete darkness. One percent paraformaldehyde was used for fixing. Samples were run on a FACSCanto II flow cytometer (BD Biosciences). Scans were evaluated for the cell concentrations. Specific gating techniques were as follows. Forward and side-scatters were set, and activated monocytes were assessed based on these assessments by mapping CD14 events, and the percentage expressing CD16 as a double-positive occurrence. Numerous washes were performed in between antibody staining to prevent false-positive staining by antibodies. Antibodies were randomly skipped to ensure specificity of the antibodies.

### Cytokine assay in the peripheral plasma

Assays were performed in triplicates and pooled results were used for the analytical assays. Interleukin-6 (IL-6) and interleukin-10 (IL-10) were assayed by the aid of Milliplex™ MAP high sensitivity human cytokine kit (Millipore). Luminex-200 fluorescent plate reader was used (Luminex). IL-6 was representative of the pro-inflammatory cytokine and IL-10 was the group representative of the peripheral cytokine that promotes differentiation of the anti-inflammatory M2 macrophages in the tissue setting.

### Conversion of peripheral blood monocytes to M1/M2 macrophages and maintenance in culture

For inducing macrophage differentiation from the peripheral blood monocytes, the donor blood was perfused through apheresis filter and prepared for magnetic separation using anti-CD14-microbeads or/and anti-CD16-microbeads (Miltenyi Biotec). Cell culture was maintained in RPMI media supplemented with 10% fetal calf serum. Liberal antibiotics were added and cultures were frequently assessed for mycoplasma infections. Granulocyte-macrophage colony-stimulating factor (GM-CSF) was used to stimulate to convert cells to a M1 phenotype (culture maintained for 7 days) and 30 ng/ml IL-10/50 ng/ml GM-CSF for conversion to M2-macrophages. Cells were viewed frequently during culture and maintained under optimal conditions. Differentiated macrophages were used for further studies. The differentiated macrophages were stained with Cd11a/b and CD206 respectively by fluorescence assay and showed high levels of purity for the M1 and M2 macrophages respectively (results not shown).

### β-Endorphin release assay by cultured M1/M2 macrophages

M1/M2 macrophages were individually stimulated with 15 μM ionomycin for 30–120 min and used for the measurement of opioid peptide. After stimulation, M1 and M2 macrophages were added to ELISA buffer followed by cycles of five freeze (20–30 s)–thaw (90–120 s) using liquid nitrogen and a warmed-up water bath. The pellets were removed and the supernatant was carefully aspirated and thereafter the opioid peptide was assayed. β-Endorphin was assayed by radioimmunoassay (RIA; Phoenix Pharmaceuticals). Protease inhibitors were used throughout the assays (kept on cold always). Additionally, calcium chelators were used as negative controls to confirm the sensitivity of the assays.

### Western blotting

For detection of protein signals, macrophage (M1) cell culture lysates were electrophoresed on 8% PAGE gels. Initial pilot experiments were done to optimize the time and voltage of separation and each blot run several times to acquire the optimal conditions. After electrophoresis, gels were transferred overnight on to PVDF membranes by running the transfer slowly at 30 V. The immunoblots were developed with 1 in 50–250 dilutions of the antibodies, again with extensive pilots performed to optimize the concentrations, as well as that of the secondary antibodies (primary antibody obtained from Abcam). Chemiluminescent signal was developed after incubation with the ECL System (GE HealthCare) and signal was developed in the dark room using Kodak X-ray.

### RNA isolation and reverse transcription

Total RNA was extracted from the lysates incorporating RNeasy Mini kit (Qiagen). All reactions were performed in cold. Complementary DNA was reverse transcribed using the QuantiTect Reverse Transcription kit (Qiagen). The experiments were always performed in nuclease free conditions to ensure the highest quality of the nucleic acid.

### Real-time-PCR to detect expression level of β-endorphin mRNA

Real-time quantitative PCR was performed using ABI 7000 SDS with the Taqman probe mixture (Applied Biosystems). Probes/primes for the analysed genes related to β-endorphin messenger expression were designed with Primer Express 2.0 software. The oligonucleotide probes were tagged with the reporter dye 6-FAM at its 5’-end and the quencher TAMRA dye at the 3’-terminal side. The internal control was chosen as the housekeeping gene actin. The PCR reaction was performed with the routine protocol of 45 cycles. The relative expression levels of the gene were compared.

### Statistics

Data were expressed as means ± S.E.M. (standard error of measurement). Paired samples were compared by student's *t* test. Analyses of variance were performed to compare means between multiple groups, as well as Tukey's post-hoc honestly significant difference (HSD) test performed to confirm robustness of comparison.

## RESULTS

### Significantly elevated levels of CD16^+^(CD14^+^) pro-inflammatory monocytes in peripheral circulation of subjects with low back pain

**Figure 1:**
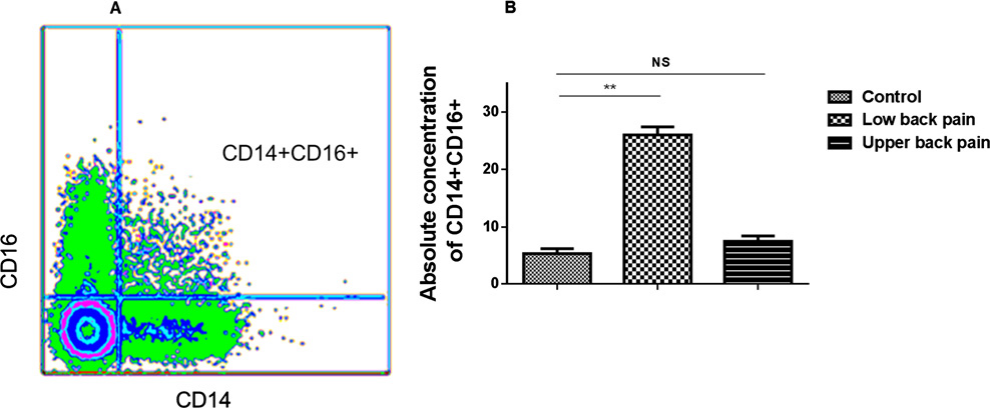
Fluorescence-activated cell sorting (FACS) image of gating strategy and histogram depicting cumulative data from all samples of the significant increase in absolute concentration of CD16 monocytes in PBMC of subjects with low back or upper back pain compared with controls (**A**) Flow image showing the gating strategy for isolation of CD16 positive cells from the pool of CD14 monocytes. (**B**) Histogram depicting cumulative data from all samples of the significant increase in absolute concentration of CD16 monocytes in peripheral blood of subjects with low back pain or upper back pain in comparison with controls. The differences between the means were highly significant.

Monocytes are classified as classical pro-inflammatory CD14^+^CD16^+^ and CD14^+^CD16^−^ monocytes, which play key anti-inflammatory role. Normally, CD14^+^CD16^+^, as opposed to CD14^+^ non-expressing CD16 classical monocytes, represent only a minor fraction of the total monocytes in the peripheral circulating blood but increase in various disease processes that have inflammation has a major basis of the disease pathophysiology. As a first step to evaluate whether the circulating pro-inflammatory monocytes may be assessed in the peripheral blood with subjects with chronic low back pain, special gating protocols were adapted to evaluate the absolute concentration of the pro-inflammatory monocytes. A representative fluorescence-activated cell sorting (FACS) image of gating strategy is shown in Figure 1(A). In comparison with control subjects, subjects with low back pain had consistently elevated absolute concentrations of the CD16+(CD14+) cells (*P*<0.01, 2-tailed *t* test), however, subjects with upper back pain did not show significant changes of the CD16+(CD14+) cells (Figure 1B).

### Anti-inflammatory cytokine IL-10 decreased in peripheral circulation of subjects with low back pain, whereas pro-inflammatory cytokine IL-6 is increased in plasma

**Figure 2:**
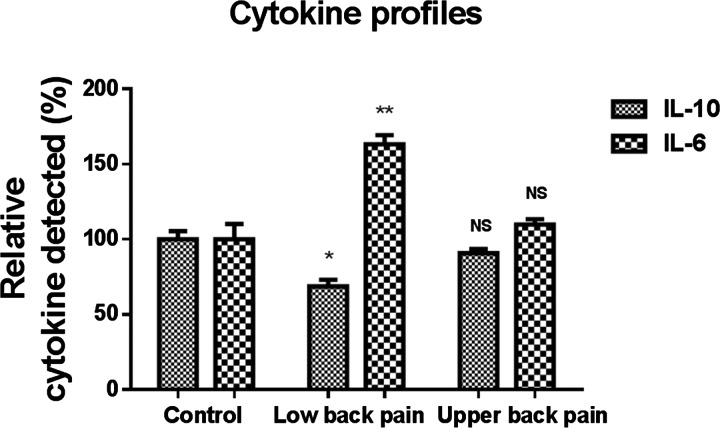
Cytokine samples showing increase in expression of IL-6 in subjects with low back pain, and decrease in the anti-inflammatory cytokine IL-10 Cytokine assays indicate imbalance of pro- and anti-inflammatory cytokines in the peripheral blood samples.

In comparison with control subjects, all subjects (individually as well as cumulatively) demonstrated significantly lowered levels of IL-10 in the peripheral circulation. IL-10 is a peripheral cytokine that plays a definitive role of conversion of monocytes to M2 class of anti-inflammatory macrophages after recruitment to tissues. Though we could not obtain DRG biopsies due to pragmatic reasons, this assay of the peripheral cytokine provides an indirect surrogate assessment of the potential of the CD14 exclusive monocytes to undergo neuro-immune transformations after being recruited to tissues. The levels of difference were significant when cumulative means were assessed for statistical differences (*P*<0.01, Student's *t* test) (Figure 2). On the other hand, the concentrations of IL-6 were significantly elevated in the plasma samples obtained from subjects with low back pain (*P*<0.05, Student's *t* test) (Figure 2). Moreover, compared with control group, subjects with upper back pain did not show significant changes of IL-10 or IL-6.

### Reduced secretion of β-endorphin by M2 macrophages cultured from subjects with low back pain

**Figure 3:**
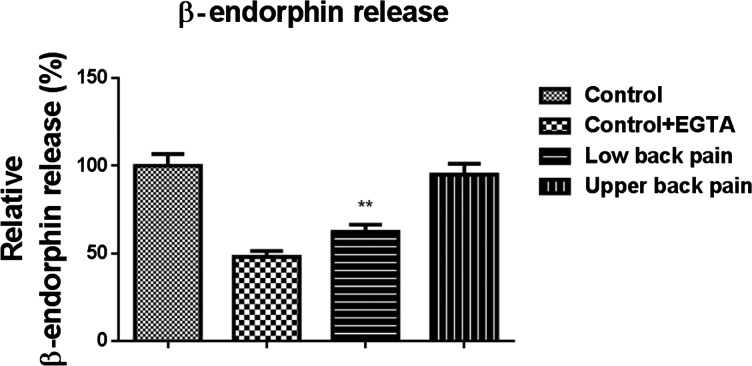
RIA showing impaired secretory capacity of β-endorphin in cultured M2 macrophages obtained from subjects with low back pain but not upper back pain Comparisons are made with control subjects. Stimulation with ionomycin was impaired when cells were incubated with the calcium chelator EGTA. The means were statistically significant when means were compared by analyses of variance. The macrophages were differentiated by controlled stimulation of monocytes *ex vivo*.

M1 macrophages showed only scanty levels of β-endorphin secretion. In contrast, M2 macrophages for control subjects showed robust secretion of β-endorphin upon ionomycin stimulation for variable periods of time. However, this secretory capacity was significantly and markedly attenuated in subjects with low back pain [cumulative data, *P*<0.001, Student's *t* test, control compared with low back pain; *P*<0.001, ANOVA with Tukey's post-hoc HSD test, multiple comparisons made between low back pain, control samples and control samples after calcium chelator ethylene glycol tetraacetic acid (EGTA) incubation; M1 secretions were not assayed for comparisons due to undetectable levels in several samples] (Figure 3). Consistently, we did not detect the differences of β-endorphin secretion between control group and subjects with upper back pain (Figure 3).

### Reduced expression of β-endorphin mRNA in peripheral blood mononuclear cells cultured from subjects with low back pain

**Figure 4:**
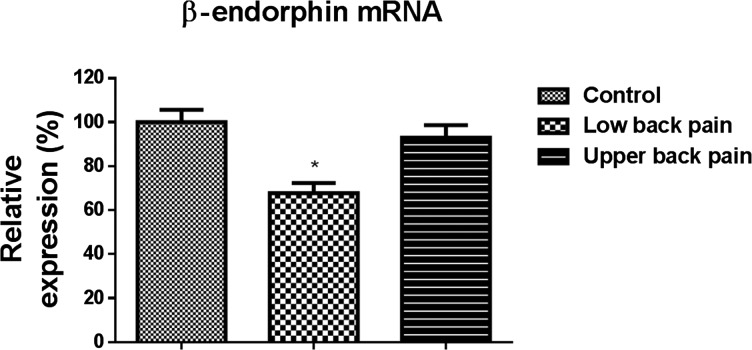
Real-time RT-PCR demonstrate significant decrease in β-endorphin mRNA expression in peripheral blood cells in subjects with low back pain but not upper back pain compared with control cells

Real-time PCR demonstrated significant reduction in β-endorphin mRNA in PBMCs obtained from subjects with low back pain, but not subjects with upper back pain in comparison with healthy controls (*P*<0.01, Student's *t* test) (Figure 4). These observations supplement the fact that there is a global genomic suppression of the transcription for endogenous opioid peptides. This global suppression of endorphin synthesis probably also explains the down-regulation of the opioid peptide expression observed when differentiated macrophages were stimulated *in vitro* in subjects with low back pain.

### Reduced expression of Dragon (repulsive guidance molecule b, RGMb) in M1 macrophages cultured from subjects with low back pain

**Figure 5:**
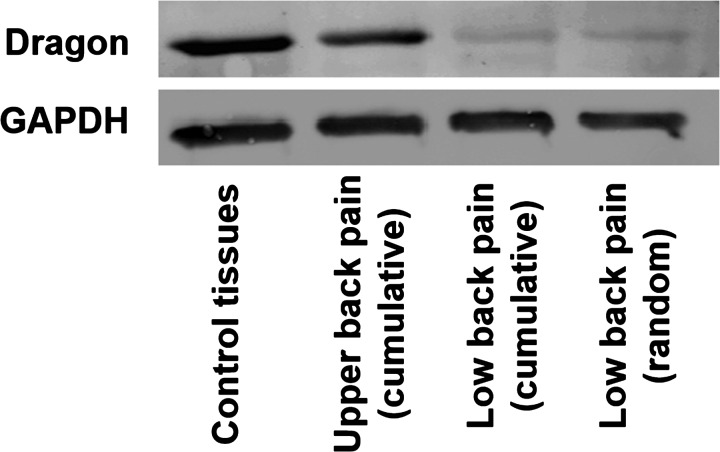
Western blotting showing diminished expression of the repulsive guidance molecule dragon in M1 macrophages obtained in subjects with low back pain compared with control tissues Cumulative patient samples, as well as a randomly chosen subject are shown. Dragon is a major negative transcriptional regulator of IL-6 expression. These findings support the view of chronic neuroinflammation as a pathogenetic basis for low back pain.

We examined the expression of dragon, a key molecule that suppresses IL-6 expression. We hypothesized that this is the major transcriptional regulation of inflammation in the M1 macrophages. In conformation to our hypothesis, dragon expression was significantly reduced in M1 macrophages obtained from subjects with low back pain, in comparison with healthy controls and subjects with upper back pain. A representative western blot along with loading controls is shown in Figure 5.

## DISCUSSION

The results of the present study provide several novel evidences regarding the mechanisms of neuro-immune inflammation that occurs with low back pain. Namely, we demonstrate that the proportions of CD16 monocytes are increased in their absolute expression in the peripheral circulation of subjects with low back pain. Furthermore, we demonstrate that the peripheral circulation of IL-10 is decreased, whereas the proinflammatory cytokine IL-6 is elevated.

Our subjects with chronic low back pain had their pain for at least 1 year, and without any other evidence of any disease whatsoever. In order to assess the molecular basis of the elevation of IL-6 and decrease in peripheral plasma levels of IL-10, we performed an *in vitro* analysis after culturing the monocytes *in vitro* and converting them to M1 pro-inflammatory or M2 anti-inflammatory macrophages by specific growth factor conditions. First, we demonstrate that the M2 macrophages have significantly diminished capacity of secretion of the opioid peptide β-endorphin. Using peripheral blood cells, we demonstrate that the mRNA expression of β-endorphin is decreased significantly in subjects with low back pain. Thus, it is likely that transcriptional blockade is operant for release of endogenous peptides, in addition to increase in pro-inflammatory monocytes. Because of pragmatic issues related to obtaining DRG biopsies, we demonstrate these aspects of alteration of balance between pro- and anti-inflammatory environment in the peripheral blood, and cells and differentiated macrophages. Surprisingly, the tempting hypotheses may be advanced that the assessment of these components may serve as reliable peripheral markers of low back pain, viz (i) assessment of CD14 and CD16 monocytes, (ii) assessment of IL-10 and Il-6 balance, (iii) peripheral differentiation of M1/M2 macrophages and transcriptional regulators like dragon and (iv) endogenous opioid peptide release.

Finally, we demonstrate that the expression of Dragon, a key repulsive guidance molecule that negatively regulates IL-6 expression [12], is manifested in M1 macrophages obtained by differentiating peripheral cells from subjects with low back pain. This indicates that a wider pro-inflammatory environment is existent in low back pain subjects. The operant cause may be mechanical factors supporting the lower back, but it remained beyond the scope of the present study, whether these molecular factors are also responsible for degenerative bone or ligament architecture changes. It could also be possible that mechanical factors resulting in compression of nerve roots may result in the neuroinflammation, and which is manifested in cellular and cytokine changes in the peripheral circulation as demonstrated in the current study.

## Abbreviations

DRG: dorsal root ganglion
HSD: honestly significant difference
IL-10: interleukin-10
PBMC: peripheral blood mononuclear cell
RGMb: repulsive guidance molecule b
RIA: radioimmunoassay
RPMI: Roswell Park Memorial Institute
